# Periurethral Abscess in a Neonate

**DOI:** 10.21699/jns.v6i3.561

**Published:** 2017-08-10

**Authors:** Umesh Bahadur Singh, J K Mahajan

**Affiliations:** Department of Pediatric Surgery, Postgraduate Institute of Medical Education and Research, Chandigarh India 160012

**DEAR SIR**

The male urethra is lined with stratified or pseudostratified columnar epithelium, which can be readily infected by N. gonorrhoeae and C. trachomatis [1,2]. A periurethral abscess is a formation of tender inflammed area in the perineum, or under the shaft of penis in the paraurethral glands of Cowper and/or Litre’s. These are usually caused by the gonococci to begin with; which are rarely isolated from the pus sample as abscesses soon become secondarily invaded with other pathogens [3]. Cowper gland’s abscess is frequently diagnosed as “syringocele”, however Litre’s gland abscess is very rare. Periurethral abscesses may be complicated by urethral fistula, stricture, and necrotising fasciitis [4]. Periurethral abscess is a rarity in neonates.

 A 20-day-old full term male baby, born to 1st gravida mother was delivered vaginally. He presented to pediatric emergency department with complaints of fever, lethargy, poor feeding and weak urinary stream since last 5 days. Antenatal scans did not show any congenital malformations. Perinatal course was also uneventful. The baby cried soon after birth and passed urine and meconium on day 1 of life. After 5 days, the baby started crying excessively during micturition and had occasional fever, although the urinary stream was good. Patient was administered antibiotics at a peripheral centre but the urinary complaints did not resolve and swelling appeared on the undersurface of penis. The baby was referred to our centre. On examination, there was a 2x3 cm fluctuent swelling at the base of penis on ventral surface in the interscrotal area and another 3x3cm swelling was seen at the root of scrotum near the perineum (Fig.1a,1b). Both the testes could be felt separately and the scrotal sacs were normal. Ultrasonographic examination revealed two periurethral collections in relation to the penile and bulbar urethra. The baby underwent perurethral catheterisation for urinary diversion which could be done easily. Needle aspiration of the swellings showed pus which were subsequently drained with small stab incisions at both the locations. Culture examination of the pus showed growth of E Coli organism. Fever subsided within 24 hours of pus drainage and the urinary catheter was removed after 3 days, once the area showed complete disappearance of the swellings. Patient passed urine in satisfactory stream and was discharged on oral medications as per the sensitivity report. At follow up visit after 1 month, a micturating cystourethrogram was performed which revealed smooth outline of anterior and posterior urethra without any outpouching or obstruction. Maternal screening for gonococcal and chlamydia infection was also carried out, which was found to be negative. At present, the patient is asymptomatic and urinary stream is good.

**Figure:**
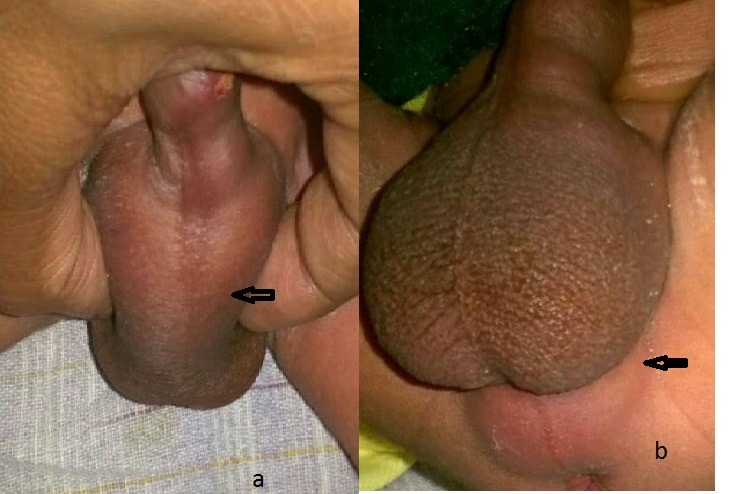
Figure 1: Periurethral abscesses around penile urethra in scrotal area (a) and perineum (b).

 A relatively high number of sexually transmitted pathogens are isolated in sexually active individuals while urinary pathogens are found more frequently in older men [5]. Isolation of sexually transmitted pathogens is rarely achieved as most of the times superadded infection with other pathogens ensues. Subepithelial progression of an infection may lead to periurethritis, infection of the periurethral glands, and abscess formation. Predisposing factors to this event include a history of gonorrhoea, previous abscesses, and urethral stricture. If the abscess penetrates Buck’s fascia, a necrotising fasciitis with extensive tissue destruction may occur. It has been postulated that the pathogenesis of periurethral abscess may evolve into a urethral stricture with subsequent urethral disruption and extravasation of infected urine [5]. Rarely, a urethral carcinoma may be implicated in the pathogenesis [3]. Treatment consisted of abscess drainage, suprapubic urinary diversion and intravenous antibiotic therapy [5].

 This report describes periurethral abscess in a neonate and the possibility of vertical trasmission of infection to a neonate during vaginal delivery.

## Footnotes

**Source of Support:** None

**Conflict of Interest:** None
